# Impact of disruptions to routine vaccination programs, quantifying burden of measles, and mapping targeted supplementary immunization activities

**DOI:** 10.1016/j.epidem.2022.100647

**Published:** 2022-12

**Authors:** Natalya Kostandova, Stacie Loisate, Amy Winter, William J. Moss, John R. Giles, C.J.E. Metcalf, Simon Mutembo, Amy Wesolowski

**Affiliations:** a Department of Epidemiology, Johns Hopkins Bloomberg School of Public Health, Baltimore, MD, USA; b Department of Epidemiology and Biostatistics, University of Georgia, Athens, GA, USA; c Department of International Health, Johns Hopkins Bloomberg School of Public Health, Baltimore, MD, USA; d Institute for Health Metrics and Evaluation, University of Washington, Seattle, WA, USA; e Department of Ecology and Evolutionary Biology, Princeton University, Princeton, NJ, USA; f Princeton School of Public and International Affairs, Princeton University, Princeton, NJ, USA; g Ministry of Health, Government of the Republic of Zambia, Lusaka, Zambia

**Keywords:** COVID-19, Disruptions, Measles, Outbreaks, Immunization campaigns

## Abstract

Measles is a highly transmissible disease that requires high levels of vaccination coverage for control and elimination. Areas that are unable to achieve and maintain high coverage levels are at risk for measles outbreaks resulting in increased morbidity and mortality. Public health emergencies, such as the current COVID-19 pandemic, pose a threat to the functioning of health systems by disrupting immunization services which can derail measles vaccination efforts. Efforts to bridge coverage gaps in immunization include the rapid return to fully functioning services as well as deploying supplementary immunization activities (SIAs), which are additional vaccination campaigns intended to catch-up children who have missed routine services. However, SIAs, which to date tend to be national efforts, can be difficult to mobilize quickly, resource-intensive, and even more challenging to deploy during a public health crisis. By mapping expected burden of measles, more effective SIAs that are setting-specific and resource-efficient can be planned and mobilized. Using a spatial transmission model of measles dynamics, we projected and estimated the expected burden of national and local measles outbreaks in Zambia with the current COVID-19 pandemic as a framework to inform disruptions to routine vaccination. We characterize the impact of disruptions to routine immunization services on measles incidence, map expected case burden, and explore SIA strategies to mitigate measles outbreaks. We find that disruptions lasting six months or longer as well as having low MCV1 coverage prior to disruptions resulted in an observable increase of measles cases across provinces. Targeting provinces at higher risk of measles outbreaks for SIAs is an effective strategy to curb measles virus incidence following disruptions to routine immunization services.

## Highlights

• Disruptions to routine measles vaccination services result in potential outbreaks.
• TSIR models can be used to map where measles outbreak risk is greatest.
• Low MCV1 coverage prior to disruptions impacts outbreak severity.
• Targeted supplementary immunization campaigns can mitigate measles outbreak risk.

## 1 Introduction

Despite considerable progress towards measles elimination goals, recent years have seen a resurgence of measles virus transmission in many areas of the globe (Durrheim et al., 2019). Given the high transmissibility of measles virus, suppressing transmission requires achieving and maintaining high levels of population immunity. The World Health Organization (WHO) currently recommends measles vaccination coverage above 90% for both the first dose of measles-containing vaccine 1 (MCV1) and the second dose of measles-containing vaccine 2 (MCV2), but many in sub-Saharan Africa are below this threshold (Durrheim et al., 2019, Poland, 2018, World Health Organization, 2012, World Health Organization). Many countries provide vaccinations through both routine immunization services, the regular vaccination of children on a defined schedule at health centers, and periodic supplementary immunization activities (SIAs) for a particular vaccine (often MCV or polio). In the majority of settings, routine vaccinations are consistently available, whereas mass vaccination campaigns (such as SIAs) are often deployed nationally over a short time frame (e.g., two weeks) and aim to vaccinate all eligible children within defined ages, regardless of previous vaccination history (Portnoy et al., 2018, World Health Organization, 2020). In many settings, achieving the WHO recommended routine coverage requires both vaccination strategies with SIAs intended to catchup children who missed routine vaccines or did not mount a protective immune response. Global coverage for MCV1 has plateaued at 85% and many countries require SIAs to bridge coverage gaps (Portnoy et al., 2018, World Health Organization, 2020). However, SIAs are resource intensive and require careful planning and mobilization efforts to vaccinate the large target population including hard-to-reach persons (Clarke et al., 2019, Cutts et al., 2021, Portnoy et al., 2018). Given the high coverage needed to interrupt measles virus transmission, even small disruptions to vaccination programs can increase risk of a measles outbreak and derail progress towards measles control and elimination.

Public health emergencies often result in diversion of resources and reduced capacity to maintain both routine programs and supplementary campaigns. For example, vaccination campaigns for measles, human papillomavirus (HPV), and polio were paused during the 2014 Ebola outbreak in West Africa (Clarke et al., 2019). Measles vaccination coverage decreased from 74% in 2013 to 58% in 2014 and one Guinea prefecture subsequently experienced an outbreak of 702 measles cases after the Ebola outbreak from January 1 to June 30, 2015 (Clarke et al., 2019, Sun et al., 2017). On March 26, 2020, the WHO called for the temporary suspension of SIAs in response to the COVID-19 pandemic (World Health Organization, 2020a). However, given the dynamic nature of the pandemic, the WHO has since provided recommendations regarding the resumption of mass vaccination campaigns that are contingent on COVID-19 burden, allowing some countries to resume vaccination efforts (World Health Organization, 2020b). Fear of exposure to SARS-CoV-2 and the shift of resources to the pandemic response raised parental concerns of attending health facilities, according to a study conducted at pediatric hospitals in Italy in March 2020 (World Health Organization, 2020a, Lazzerini et al., 2020). Contracting COVID-19 through attending health facilities or contacts with vaccinators is a primary argument for suspending immunization programs during the current pandemic (Abbas et al., 2020). However, early evidence suggests that the potential risk of contracting COVID-19 at vaccination sites is far outweighed by the risk of disease outbreaks from temporary suspensions of immunization services. In Africa, the predicted number of measles-attributable deaths averted by maintaining measles vaccination for 6 months during the COVID-19 pandemic is 10,282 deaths with an associated 751 excess COVID-19 deaths (Abbas et al., 2020). Thus, the evidence supports sustaining vaccination programs during the pandemic.

Despite recommendations to maintain vaccination efforts during the pandemic, there were global disruptions to routine immunization services (Abbas et al., 2020, Causey et al., 2021). Further, the WHO suggested the temporary suspension of mass vaccination campaigns (World Health Organization, 2020a, Abbas et al., 2020, Causey et al., 2021). Among the countries with reduced vaccination levels in May 2020, the largest proportion were from the WHO African Region (World Health Organization). For example, in Kenya, disruptions to routine immunization services were estimated to be as high as 33%, and a previously planned SIA was postponed (Mburu et al., 2021). The WHO has since created a framework to guide decisions regarding the resumption of planned mass vaccination campaigns despite the ongoing pandemic (World Health Organization, 2020). However, once the decision is made to resume or implement an SIA, questions remain on how to effectively mobilize efforts. SIAs require resources that may be difficult to secure during public health emergencies such as personnel, vaccine doses, training, and cold chain management (Sibeudu et al., 2020). Efforts to deploy effective and resource-efficient SIA campaigns that optimize factors such as timing and location to prioritize high-risk communities are vital for sustaining low measles burden. Ideally, prioritization of SIAs should be in areas where there are more susceptible persons and the greatest risk of outbreaks, but identifying these areas and informing SIA mobilization can be difficult in low-resource settings. Understanding how to mitigate outbreak risk using SIAs is key for countries to maintain low measles burden during public health emergencies, such as the current COVID-19 pandemic.

We investigated disruptions to routine vaccination services, subsequent outbreak risk, and SIA strategies in Zambia, a country of roughly 17.8 million persons in southern Africa (The World Bank, 2022). Since 2010, there have been few reported cases of measles and overall high routine coverage of MCV1, with a national average of 90% (Carcelen et al., 2022, Katemba et al., 2019). However, despite high coverage, Zambia has been subject to measles outbreaks (Katemba et al., 2019). For example, the country experienced a large outbreak in 2010/2011 resulting in 35,572 measles cases and 242 measles deaths. The COVID-19 pandemic has disrupted health services throughout the country since the first COVID-19 cases were identified on 18 March 2020 (Zambia National Public Health Institute, 2020). To date (as of October 3, 2021), there have been more than 212,000 total cases of COVID-19 in Zambia with the most populous provinces, Lusaka and Copperbelt, reporting the highest number (Zambia National Public Health Institute, 2021). However, it is hopeful that measles morbidity and mortality among children in Zambia will be avoided due to the implementation of two successful Child Health Week mass vaccination campaigns that took place from June 22–27, 2020, and November 23–28, 2020 (Babu et al., 2021, World Health Organization - Zambia, 2020, Zambia National Public Health Institute, 2021). It is imperative to evaluate SIA campaigns as a mechanism to close vaccination gaps in children who may have missed measles vaccination during the pandemic. We used a spatial transmission model of measles dynamics for districts in Zambia to estimate the possible impact of interruptions to immunization services and the effectiveness of national and sub-national SIA strategies. We identified spatial risk patterns of outbreaks in the near and long term to identify the effectiveness of various SIA strategies to mitigate a measles outbreak during public health emergencies and disruptions to routine services.

## 2 Materials and methods

### 2.1 Population data

**Fig. 1:**
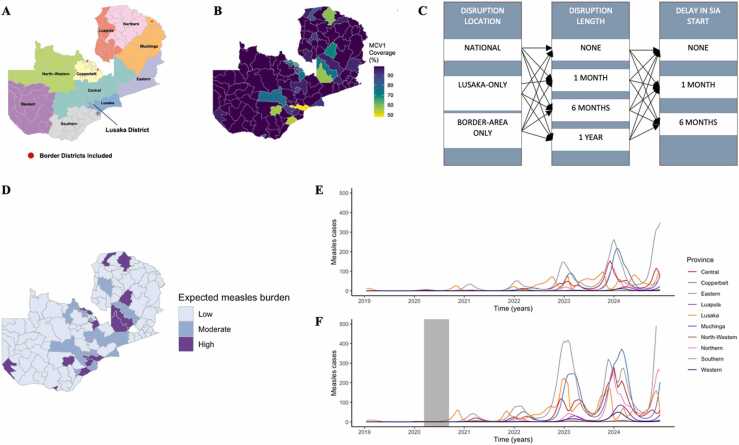
**Overview of scenarios and illustration of the risk of measles burden due to disruptions to routine MCV1 services. (A)** Map of Zambia with provinces labeled. **(B)** Routine MCV1 coverage in Zambia by district using 2018 estimates. **(C)** Scenarios run in this analysis. Each disruption location was run for varying lengths and addressed with different SIA strategies. **(D)** District-level distribution of measles burden from January 2020 to December 2022 following disruption to routine MCV1 services lasting six months not followed by an SIA. **(E)** Time-series of province-level distribution of measles burden in control scenarios with no disruptions and **(F)** a six month national disruption to routine MCV1 services not followed by an SIA. Shaded area represents the period of disruption.

Zambia is divided into ten provinces and subdivided into 117 districts; however, we modeled measles dynamics across 115 districts since two districts (Chilinda and Lusangazi) were not included in the most recent census or vaccination coverage data used in this analysis (Fig. 1 A). Lusaka Province includes the national capital and is the most populated province with 2.63 million persons in 2019 (Zambia Statistics, 2020). Demographic estimates of population size, birth rates, and death rates used in this analysis included district-level census data from 2011 and projected to 2035 and are publicly available at https://www.zamstats.gov.zm/. Spatial data used for generating maps are publicly available at https://zambia-open-data-nsdi-mlnr.hub.arcgis.com/.

### 2.2 Vaccination data

District-specific routine MCV1 vaccination coverage from 2013 to 2018 was estimated from administrative data provided by Zambia’s EPI office via the Zambia Health Management Information System (HMIS). Coverage was estimated by dividing the total number of administered MCV1 doses in a year by the target population or 0.4% of the mid-year population (assuming a crude birth rate of 40 per 1000 population not accounting for infant mortality). MCV1 coverage estimates over 100% were capped at 99% to better estimate routine as opposed to the coupled routine and supplemental programs. To simulate measles dynamics from 2016-2025, the coverage level in 2018 were used as a proxy.

### 2.3 Simulating measles transmission dynamics

A spatial semi-mechanistic discrete time-series SIR (TSIR) model was used to simulate measles virus transmission dynamics for districts within Zambia. Previous reports suggest TSIR models are sufficient for simulating measles dynamics as they allow for variability of parameters such as births and vaccination coverage at different time points (Grenfell et al., 2002). Discrete periods were defined as two-week intervals to account for the two week period between measles virus infection and recovery (one time step = two weeks) (Finkenstädt BF, 2000). The susceptible population per time step was estimated by births, population sizes, and routine vaccination coverage for each district. The initial susceptible fractions were set to province-level seroprevalence estimates from biospecimens collected during 2016 Zambian Population HIV Impact Assessment (Carcelen et al., 2022). Connectivity data were estimated using a gravity model fit to Namibia, a neighboring country in sub-Saharan Africa, and scaled with population sizes and distance reflective of districts within Zambia (Carcelen et al., 2022, Giles et al., 2021). This model was used to simulate measles dynamics, interruptions to routine vaccination programs, and strategies for SIAs. The COVID-19 pandemic was used as a framework to guide scenarios on reductions to routine immunization services and the possible impact of SIA campaigns on mitigating measles outbreaks.

The numbers of births and deaths at each time step were estimated using census data as described above. Simulations were run from 2016 to 2020 (prior to disruptions) to ensure stable measles dynamics. We ran simulations for an additional five years post-disruptions to obtain future projections of measles cases. Scenario outputs were simulated from an average of 100 runs for each simulated scenario. In a TSIR model, the transmission coefficient, β_0_, can be estimated by using the reproductive number (R_0_) as a proxy, which we are assuming to be 15 based on recent reports (Guerra et al., 2017). The coefficient addressing the variation of amplitude, β_1,_ is assumed to be moderate at 0.3 (Ferrari et al., 2008). Both of these values inform the time-varying seasonal forcing coefficient, β_t_ (Finkenstädt BF, 2000).

The number of additional children susceptible (births that remain unvaccinated after routine immunization) at any given time step ($\theta_{i,t}$) and the overall change in susceptible children in the population ($S_{i,t}$) is estimated by:

$$\theta_{i,t}=\mu_{i,t}*\left(1-\left(\rho_{i,t}*V_{i,t}\right)\right)$$

$$S_{i,t}=\left(\left(1-\sigma_{i,t}\right)*S_{i,t-1}\right)-I_{i,t}+\theta_{i,t}$$

Here, $\rho_{i,t}$ is the proportion of routine immunization services that will remain without interruption at any given time step in each district. If no disruptions are assumed, this value will be one and there will be no additional susceptible persons as a result of disrupted immunization services. *V*_*it*_ represents routine vaccine coverage estimates (multiplied by vaccine effectiveness) and $\mu_{i,t}$ is the number of new births per district per time step. The proportion of persons reached during an SIA is represented by $\sigma_{i,t}$.The number of children infected at any given time step is estimated by:

$$\beta_{t}=\beta_{0}\left(1+\beta_{1}*\cos\left(2*\pi *t\right)\right)$$

$$E\left[I_{i,t+1}\right]=\frac{\beta_{t}I_{i,t}S_{i,t}^{\alpha }}{N_{i,t}}$$

$$I_{i,t+1}∼\mathrm{NegBin}\left(E\left[I_{i,t+1}\right],I_{i,t}\right)+\sum_{j}\mathrm{Binom}\left(I_{j,t,}M_{i,j}\right)$$

The mixing parameter, α, has been reported to be less than but close to one to account for scaling down of the well-mixed assumption with discrete time steps (Glass et al., 2003). We set α to 0.975 (Glass et al., 2003). $M_{\mathrm{ij}}$ represents the proportion of persons moving between districts and at each time step estimated by the gravity model described above. Each infected individual in location j has a probability of traveling to location i based on $M_{i,j}.\;$Given that this value is often small, we do not remove these infected individuals from j. We modeled a 2016 SIA campaign with estimated coverage of 95% (Glass et al., 2003, Hayford et al., 2019). Vaccine efficacy for one dose of measles-containing vaccine was set to 93% (CDC, 2022). For each district, we ran the model 100 times allowing process stochasticity from random draws of the negative binomial distributions. Model output is displayed for each district and at the province level; provincial level number of cases are an aggregate of cases from all districts located within a province.

We allowed for introductions of two infectious individual to Lusaka district in January of each year. Spatial connectivity among the 115 Zambian districts was modelled by fitting a gravity model to Namibia Call Data Records (CDRs) collected in Namibia from October 2010 to April 2014, which have been described in detail in other manuscripts (Giles et al., 2021). Briefly here, the gravity model formulation uses distance among locations (D) and population sizes (N) as covariates; the model is fitted to the movement matrix (M) with a Poisson likelihood link function:

$$M_{\mathrm{ij}}∼\text{Pois}\left(\pi_{\mathrm{ij}}N_{i}\right)$$

$$\pi_{\mathrm{ij}}\propto \theta \left(\frac{N_{i}^{\omega_{1}}N_{j}^{\omega_{2}}}{d_{\mathrm{ij}}^{\gamma }}\right)$$

Where $M_{\mathrm{ij}}$ are the normalized observed mean monthly number of travelers moving from origin i to destination j, scaled by 0.3 to account for lower mobility of younger children. The exponential parameters $\omega_{1}$ and $\omega_{2}$ are weights that modify the contribution of origin and destination population sizes, and θ is a proportionality constant. The denominator of the gravity model, $d_{\mathrm{ij}}^{\gamma }$, serves as the dispersal kernel function. The likelihood function was fitted to the data using Bayesian MCMC inference made available in the ‘mobility’ R package, which uses the JAGS (Just Another Gibbs Sampler) MCMC library for parameter estimation (Giles et al., 2021, Giles et al., 2020). Values of parameters and initial conditions are provided in Table S1.

### 2.4 Quantifying disruptions

We simulated the risk of measles outbreaks following national and sub-national disruptions to routine measles immunization services in three different scenarios. In all three scenarios, we assumed that routine coverage fell by 75% based on previous studies exploring disruptions during epidemics (Takahashi et al., 2015). The three scenarios we considered were: (1) national disruptions across all districts and two focused on areas of the country that reported large numbers of COVID-19 cases and had stricter non-pharmaceutical interventions in place including (2) Lusaka Province and (3) four border districts including Nakonde (Muchinga Province), Ndola and Chililabombwe (Copperbelt Province), and Chirundu (Lusaka Province) (Zambia National, 2020a). Community-wide transmission was first reported in Lusaka Province, with 34 confirmed cases by the end of March 2020 (Zambia National, 2020b). Expected measles burden was categorized as: Low = cases < 10, Moderate = 10–50 cases, and High = >50 cases occurring from January 2020 to December 2023.

*Evaluating the Impact of various SIA Strategies*: Implementation of SIA campaigns was simulated at the following times: September 2020 (no interruption; SIA implemented according to national strategy); 4 week delay; and six month delay. SIA campaigns lasted for two weeks and were assumed to achieve 75% coverage with 93% vaccine efficacy independent of prior measles vaccination status. While this coverage is substantially lower than estimated coverage in previous campaigns, it is consistent with preliminary results of September 2020 SIA (Ministry of Health Zambia, 2021). Simulated campaign scenarios were implemented only in locations that experienced disruptions. Additionally, to evaluate if targeted SIA campaigns are effective in reducing the risk of national measles outbreaks, we simulated an SIA campaign only in Lusaka Province in a scenario with national disruptions. We used our model to project measles case estimates from January 2020 to December 2023 to compare estimated burden of measles under different routine interruption and SIA delay scenarios.

All computations were done with the R statistical computing language, version 4.0.2 (The R Project for Statistical Computing,).

## 3 Results

We modeled measles dynamics in Zambia across 10 provinces and 115 districts (Fig. 1) encompassing a range of disruption scenarios by varying the duration and geographic scale of the disruptions. SIA strategies were then compared to investigate the impact on mitigating simulated measles outbreaks throughout the country (Fig. 1 C). Interruptions to routine MCV1 services resulted in a projected increased case burden after disruptions (Fig. 1D-F).

### 3.1 Impact of disruptions to routine vaccination services

Overall, routine MCV1 coverage is high in Zambia with an average coverage of 93% (range 48%−99% across districts) in 2018 although there are districts with coverage below 90% (Fig. 1B, Table 1). Furthermore, some provinces had alarmingly low levels of seropositivity against measles in 2016 (Table 1). This puts few locations at risk for an outbreak if routine services are not maintained. We simulated measles dynamics with a range of disruptions to routine services nationally, only in the capital province of Lusaka, and border areas where initial transmission of SARS-CoV-2 was detected (Zambia National, 2020b). Based on early reports of observed disruptions of a 33% reduction to routine vaccination in Kenya, we used a range of reductions (25%, 50%, or 75%) (World Health Organization,). Overall, the reduction percentage did not qualitatively change the results but impacted the overall number of estimated measles cases (Fig. S1). For each disruption, we varied the duration of the disruption to routine services (one month, six months, and one year) and assumed that vaccination coverage decreased by 75% relative to baseline. Since the focus of this study is to explore disruptions and SIA strategies, we assumed that routine coverage immediately returns to normal once disruptions end but recognize that this may not be feasible and would therefore influence case distribution (Fig. S2).

**Table 1:** **Coverage of measles-containing vaccine and measles seropositivity in Zambia, by province.** Coverage of first dose of measles-containing vaccine (MCV1) estimates (2018) acquired from the Zambia Health Management Information System (HMIS). Measles seropositivity estimates obtained from the biospecimens collected during 2016 Zambian Population HIV Impact Assessment (Carcelen et al., 2022).

Province	MCV1 coverage, 2018 (%)	Measles seropositivity, 2016 (%)
Central	90	84.7
Copperbelt	85	82.7
Eastern	99	80.5
Luapula	99	78.2
Lusaka	91	82.9
Muchinga	96	86.1
Northern	94	87.0
North-Western	99	83.2
Southern	86	81.0
Western	98	83.4

**Fig. 2:**
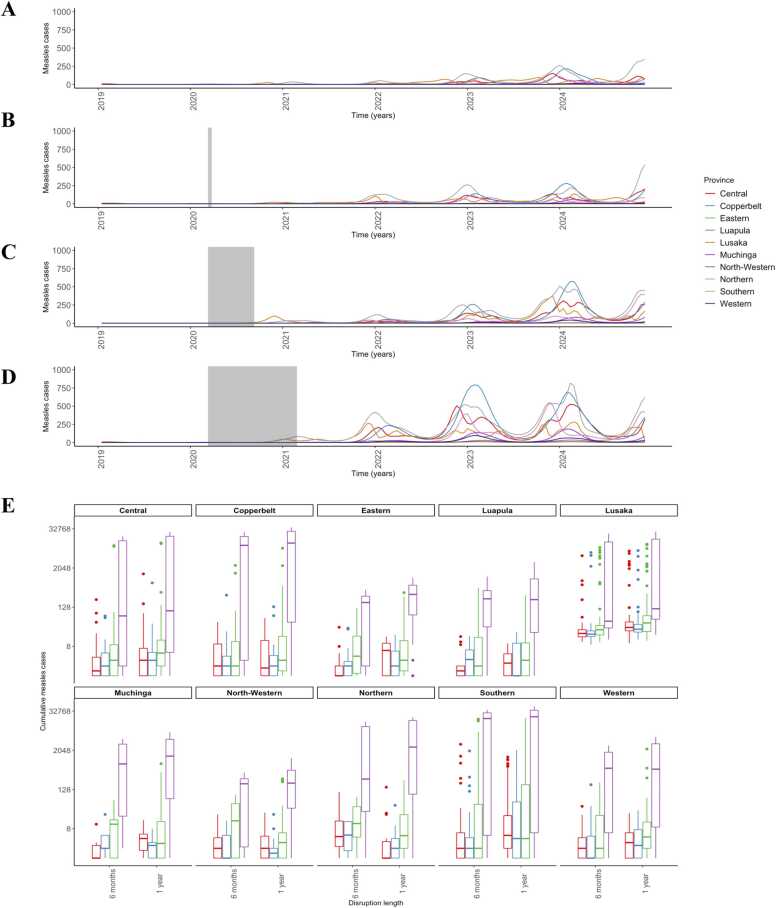
**Province-level case distribution in response to disruptions lasting one month, six months, or one year coupled with variable national SIA timing scheme.** Time series of measles burden post-disruptions not followed by an SIA with disruption length varying by (**A**) no disruption **(B)** one month **(C)** six months, and **(D)** one year. The shaded area represents the period of disruptions to routine MCV1 services. **(E)** Measles burden for all provinces from January 2020 to December 2023, for different durations of routine service disruptions and delays in implementation of 2020 SIA. Y-axis is presented on logarithmic scale for convenience.

Disruptions lasting only one month did not result in large differences in measles cases during the interruption period compared to control scenarios with no disruptions, with an average difference of one case per province (Fig. 2 A-B). However, disruptions to routine services lasting six months or more resulted in a high increase in case burden of measles.

If no SIA is implemented during the post-disruption period, there are large risks for measles outbreaks across the country (Fig. 2). The average number of cases nationwide remains low one-year post-beginning of disruptions, but quickly increases by 2022. By January 2022, the average number of cases in the six-month disruption scenario is twice that of no-interruption scenario, and the average number of cases in one-year disruption scenario is almost 6 times that of average cases in no-interruption scenario (Fig. S3). From January 2023 through December 2023, disruptions lasting six months or one year produced a predicted excess of roughly 13,800 and 34,600 measles cases compared to control scenarios, respectively. Similar to the case distribution during disruptions, the provinces with the greatest measles burden during the post-disruption period were Southern, Copperbelt, Lusaka, and Central (Fig. 2E), resulting in an excess of over 1000 measles cases each compared to control scenarios. The provinces with the lowest estimated burden of measles given disruptions lasting six months or one year were Eastern and North-Western. These provinces accounted for approximately 45 excess measles cases each during the period of 2020–2023 (Fig. 2E). Areas with the greatest predicted burden of measles cases post-disruptions had the lowest routine MCV1 coverage. Lusaka, Central, Copperbelt, and Southern Provinces had routine MCV1 coverage of 91%, 90%, 85%, and 86%, respectively (Table S1), all at or below the WHO recommended vaccination coverage for measles. Lusaka is also the most connected province in the country. Disruptions occurring only at border areas (Nakonde, Ndola, Chililabombwe, and Chirundu – see Fig. 1 A) did not result in higher expected measles burden compared to no disruption strategy (Fig. S4A, Fig. S4B). For disruptions occurring in Lusaka only, the number of expected measles cases increased until mid-2022 compared to no disruption strategy, followed by a slightly lower number of expected measles cases than in a no disruption scenario (Fig. S4A). From January 2020 to December 2022, we would expect a slightly higher number of cases of measles in Lusaka province under this scenario (Fig. S4B).

### 3.2 SIAs to address national disruptions

We investigated the impact of an SIA on reducing the excess burden of measles cases. Given that there was no clear outbreak risk for disruptions lasting one month, we only considered the impact of an SIA for disruptions lasting six months or one year. Traditionally, SIAs have been conducted nationally, as was the SIA completed in September 2020, and we first considered the impact of national SIAs. Given that national SIAs, such as the 2020 Zambia SIA, are planned well in advanced, we considered scenarios where the 2020 SIA was administered as planned (in September 2020), or with variable delay (1 month, 6 months, and 12 months). We found that a national SIA occurring one-month post-disruption performed similarly to an SIA implemented on schedule, and both resulted in a large reduction in measles burden. In both scenarios, few cases were seen outside of Lusaka province (Fig. 2E). Longer delays in implementing an SIA had, on average, resulted in higher expected cumulative case count from 2020 through 2023. For example, a 36 week delay in implementing an SIA, along with a one year disruption in routine MCV1, resulted in almost 1000 cases of measles (Fig. S5B). However, the relationship between the time an SIA was implemented and seasonality played a role in the effectiveness of an SIA. For example, waiting an additional 4 weeks to implement an SIA resulted in a lower average number of cumulative cases than implementing an SIA with a 36 week delay (Fig. S5B). Further, delaying an SIA did not produce a homogeneous effect across all provinces. As seen in Fig. 2E, depending on the length of routine disruptions, delaying an SIA may be possible in some provinces, such as Luapula, without resulting in a high expected number of measles cases. Provinces such as Central, Copperbelt, and Southern that are at higher risk for measles post-disruptions would result in a predicted excess of approximately 600, 1000, and 800 measles cases if an SIA were delayed six months compared to one-month post-disruptions lasting six months (Fig. 2E). This suggests that there may be areas that should be targeted for an SIA earlier if resources were limited and a national campaign could not be implemented in a timely manner.

### 3.3 National vs sub-National SIAs

**Fig. 3:**
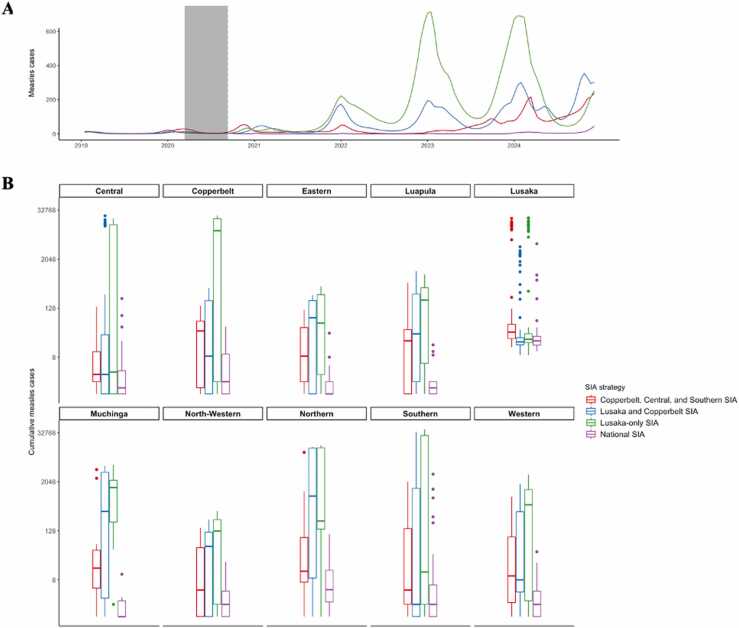
**Exploring national vs targeted SIA strategies in response to disruptions at the province-level.** Provinces targeted for SIAs were selected based on population size and outbreak risk. **(A)** Time series of SIA strategies addressing national measles burden post-disruptions lasting six months. The shaded area represents the period of disruptions to routine immunization services and the dotted line represents the 2020 SIA. **(B)** Measles burden associated with SIA strategies for all provinces from January 2020 to December 2023. Y-axis is presented on logarithmic scale for convenience.

Given differences in the outbreak risk, susceptible populations, and impact of COVID-19 on the healthcare system, we investigated the effectiveness of targeted SIAs to minimize measles outbreak risk that would require fewer resources. We investigated two strategies: prioritizing locations with the largest population sizes (Lusaka only, Lusaka and Copperbelt Provinces) or those that experienced the highest risk of outbreaks given national disruptions. National SIAs post-disruptions lasting six months prevented national outbreaks up to a year after interruptions (Fig. 3A). Different SIA strategies resulted in variable expected measles burden. Targeted SIAs only in Lusaka with a routine disruption lasting six months produced a national excess of about 14,000 measles cases compared to a national SIA over the course of 2020–2023 (Fig. 3B ). Targeted SIAs in Lusaka and Copperbelt with disruptions lasting six months produced a national excess of about 5000 measles cases compared to a national SIA over the same 4 years (Fig. 3B ). An SIA occurring only in the high-risk provinces (Central, Copperbelt, and Southern) with routine disruptions lasting six months resulted in a predicted excess of approximately 2000 measles cases over the course of the four years compared to a national SIA (Fig. 3B ). Measles burden risk was highest for SIAs only in Lusaka and lowest for SIAs occurring in high-risk provinces. Notably, targeting high-risk burden provinces maintained very low numbers of measles cases until 2023, following which number of cases rose quickly and reached incidence levels of the other strategies by mid-2024. Targeting high risk provinces may thus provide some leverage by delaying the need to implement a national campaign.

### 3.4 Mapping burden of measles cases

**Fig. 4:**
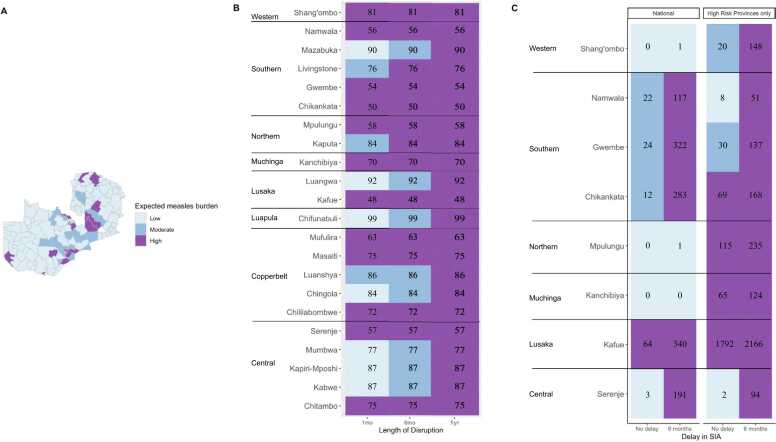
**Characterizing district-level expected burden of measles cases given varying disruptions lengths and comparing national versus targeted SIA strategies. (A)** Map of Zambia illustrating expected measles burden across districts post-disruptions lasting six months not followed by an SIA. **(B)** Distribution of district-level expected measles burden following disruptions of varying lengths of one month, six months, or one year. Only districts with “High” expected burden following 1 year of routine disruptions are included. Numbers in cells are the 2018 estimated MCV1 coverage. **(C)** District-level distribution of expected measles burden following varying SIA strategies and routine MCV1 disruptions lasting six months. Only districts with “High” expected measles burden following 6 months of routine disruptions (either under national or High-Risk Provinces only SIA strategy) are displayed. Numbers in cells indicate cumulative number of cases between January 2020 – December 2023. The following values were used to categorize outbreak risk: Low = cases < 10, Moderate = 10–49 cases, High = >50 in January 2020 – December 2023 period.

These overall patterns mask substantial finer-spatial heterogeneity in risk. Given the varying routine coverage values, populations at risk, and introduction events, we further explored district-level outbreak risk. We considered a simulation with a six-month disruption since this is likely the most consistent with the actual impact in Zambia. Using this disruption duration, we then assigned each district a level of burden based on the estimated number of cases from January 2020 to December 2023. Overall, there was substantial heterogeneity in expected burden of measles across districts (Fig. 4 A). Most districts with “High” risk of an outbreak (caseload greater than 50) are from Central, Copperbelt, and Southern Provinces (Fig. 4 A).

Unsurprisingly, risk varied based on the duration of the disruption, with longer disruptions resulting in a larger number of districts at a higher outbreak risk (Fig. 4B). However, the majority of districts remained in the same risk category (i.e. those who were at low risk remained at low risk regardless of the disruption length). There were 22 districts that reached “High” estimated burden at least for one of the scenarios with varying routine disruption lengths (Fig. 4B). Despite disruptions lasting one month producing low expected burden in most districts, there were 12 districts with “High” expected burden with short interruption lengths; among these districts, routine MCV1 was very low (Fig. 4B). Districts with higher MCV1 coverage had, with some exceptions, higher expected caseload compared to districts with low coverage following disruptions of any length (Fig. 4B). The districts with low routine coverage were less likely to have high measles burden unless the length of routine disruptions reached 1 year; Mazabuka, Luangwa, and Chifunabuli districts, which had vaccination coverage 90% or higher in 2018, had “Low” burden of measles with 1 month disruption of routine MCV1; “Moderate” with 6 months disruption, and “High” with 1 year of routine disruptions. Districts with low MCV1 coverage, such as Kafue (48%), Chikankata (50%), and Gwembe (54%) had high measles burden under all three scenarios considered (Fig. 4B).

SIA strategies addressing disruptions of six months show reduced burden in both national and targeted SIAs (Fig. 4 C). National SIAs had the largest reduction in measles burden, but a targeted SIA occurring only in high-risk areas is comparable except for a few districts. Only eight districts (Shang’ombo, Namwala, Gwembe, Chikankata, Mpulungu, Knachibiya, Kafue, and Serenje) maintained high expected measles burden after an SIA targeting high-risk areas, following a 6-month delay, and only 4 had high expected burden when this SIA was implemented on time (Chikankata, Mpulungu, Kanchibiya, and Kafue) (Fig. 4 C). These results further illustrate the distribution of measles within provinces and where a targeted SIA campaign should start at a district-level.

## 4 Discussion

In times of public health crises, there are many public health concerns that need to be addressed during and after the declared end of the crisis. Importantly, responding to one crisis must not prevent the maintenance of routine health services. Given the high transmissibility of measles virus and importance of measles vaccination campaigns, countries are at a high risk of an outbreak following disruptions from the overall impact of the COVID-19 pandemic. Using a simulation model of measles virus transmission in Zambia, we investigated the impact of disruptions to routine immunization services and the effectiveness of various SIA strategies to reduce expected cases of measles. We found that if disruptions last for one month or less, the overall high routine coverage in Zambia will prevent widespread transmission even without an SIA, although for a limited time (about a year). However, interruptions lasting greater than six months pose a large risk of measles outbreaks nationally. The primary driver of where outbreaks occur is the routine MCV1 coverage prior to the disruption. If the coverage levels were already below the critical vaccination threshold for measles, then these areas are particularly susceptible to disruptions and subsequent outbreaks. Central, Southern, Lusaka, and Copperbelt Provinces had the greatest expected cases of measles following disruptions lasting six months or longer in this analysis. These provinces, with exception of Lusaka, had the lowest routine MCV1 coverage in Zambia. Mapping where the greatest burden of measles outbreak persists is critical in informing SIA strategies.

The results from this study support the use of national and targeted SIAs as effective tools for reducing outbreak risk in response to interruptions in routine immunization services. A national SIA gives rise to the greatest reduction in measles cases following disruptions but may not be feasible. Organizing and mobilizing a nationwide SIA is challenging, and attempting to do so following a public health emergency may be accompanied by more barriers. Targeted sub-national vaccination campaigns are less resource intensive and can be deployed more rapidly, which is critical for preventing measles outbreaks. Our analysis suggested targeted SIAs in Lusaka and Copperbelt, the largest provinces, reduced measles burden but did not address susceptible populations in other high-risk provinces such as Southern and Central. Directing SIAs to high-risk provinces (Central, Copperbelt, and Southern) is the most effective targeted campaign strategy in addressing measles susceptibility post-disruptions. Focusing efforts on three provinces for an SIA is more feasible than attempting a national SIA shortly after disruptions. By leaving Lusaka out of the targeted SIA, there is a risk of increased case numbers in the capital province. However, Lusaka Province has very high measles vaccination coverage and is less sensitive to disruptions to routine immunization services. Provinces and districts that have high routine coverage levels are less susceptible to outbreaks during disruptions and post-disruption periods. Still, while targeted SIAs were effective in reducing excess cases of measles during a year or two, over time, the number of susceptible individuals built up, reinforcing that a single SIA is not an effective strategy for measles control and elimination on its own.

There are some limitations to this study. We assumed that routine MCV1 coverage returns to pre-disruption rates immediately after the end of interruptions, which may be untrue. It is unclear to date the rate at which coverage returned to normal and the duration of these effects. For example, some provinces and districts may experience a slower return to regular coverage levels, which may heterogeneously impact measles dynamics across the country. Additionally, the magnitude of the actual impact on routine services during the COVID-19 pandemic remains unknown in many settings. In addition, the SIA effectiveness will vary depending on coverage levels achieved and we used a fixed value, however additional studies should be conducted to better estimate the actual coverage that was achieved. Additionally, there are concerns regarding the accuracy and quality of routine MCV1 coverage; this is key for accurately informing models like the one used in this analysis. Serological evidence, as a more direct measure of exposure and susceptibility, would help limit the reliance on MCV1 coverage and provide more realistic estimates. Incorporating results of the 2016 serological study in Zambia allowed us to refine the estimates of immunity, but more up-to-date and granular studies would surely improve this and future simulations. Finally, we do not consider the second routine dose of measles-containing vaccine (MCV2) in this analysis, which would impact both coverage and vaccine efficacy.

Efforts were made to simulate scenarios that were realistic and plausible in a setting similar to Zambia. However, in the recent past, Zambia achieved near-elimination of measles in much of the country. Given low case counts prior to 2020, averaging an annual of 14 reported cases between 2013 and 2019 (World Health Organization,), it was difficult to calibrate the model to match with realities of the country. For example, we found that evaluating the effect of a delay in SIA implementation could have counter-intuitive effects. While reducing any delays in SIA implementation is intuitive, sensitivity analysis shows that, at a certain point, carrying out the SIA prior to peak season may be more important (Fig. S5). In many settings with low measles transmission, such as Zambia, the variability in when peak transmission may occur is difficult to assess. Similarly, timing of beginning of routine MCV1 interruption period may confound the effect of duration of this interruption; however, sensitivity analysis shows that, while absolute numbers of excess cases change, the qualitative results are relatively robust (Fig. S6). Finally, we explored the potential impact of disruptions to routine systems, allowing for cases to be introduced into Lusaka district (the capital district) at beginning of each year, but cases may be introduced or imported into other locations.

Using data to map and prioritize provinces and districts where outbreaks are likely to occur given disruptions to routine immunization services to inform SIA strategies is an effective method to reduce measles burden. Not only is this informative for mapping where outbreaks are likely to occur given disruptions, but supports the need for continued efforts to achieve and maintain high MCV1 coverage estimates. However, efforts to ensure routine immunization return to normal after the end of disruptions should be prioritized. Identifying areas that were first impacted by SARS-CoV-2 was not a risk factor for large measles outbreaks and was not an effective strategy for targeting SIAs. In contrast, ranking districts of lower routine MCV1 pre-disruptions as a higher priority was a more effective strategy.

Scenarios for this study were run in Zambia, but the model and themes can be extended to other settings since globally there were disruptions to many routine services such as childhood vaccination programs. Additionally, the COVID-19 pandemic was used as a framework to estimate the magnitudes of disruptions and identify areas that may have experienced more disruptions, but the ideas presented in this study are generalizable to other public health emergencies. More generally, inferences from this research can be used to address public health emergencies by evaluating SIA strategies that will ultimately result in optimal reductions in susceptible populations and excess burden of measles.

## 5 Conclusion

National disruptions to routine immunization of more than one month pose a threat to Zambia's current control of measles transmission. Interruptions to routine services lasting one month did not have a large effect on cumulative number of cases, but disruptions lasting six months or greater resulted in an observable increase in measles transmission and cases post-disruptions. Provinces that comprised the greatest outbreak risk were those with the lowest routine MCV1 coverage (Central, Southern, and Copperbelt), with exception of Lusaka, which had high connectivity with other locations and has high population. While implementing nation-wide SIA, as was done by the Ministry of Health of Zambia, was the most effective approach to stemming nation-wide cases of measles, prioritizing high risk provinces for a targeted SIA as soon as possible is a plausible alternative in addressing susceptible populations. Mapping outbreak risk given disruptions to routine immunization is a key step in determining effective SIA strategies that are resource-efficient and capable of rapid mobilization.

## Funding

AW is supported by a Career Award at the Scientific Interface from the Burroughs Welcome Fund. NK, SL, JRG and AW are all supported by National Institute of Health Director’s New Innovator Award, grant number DP2LM013102–0. NK and AW are also supported by the 10.13039/100000060National Institute of Allergy and Infectious Diseases (1R01A1160780–01).CJEM is also supported by the 10.13039/100000865Bill and Melinda Gates Foundation (grant number INV-016091).

## CRediT authorship contribution statement

**Natalya Kostandova:** Formal analysis; Writing – original draft; Writing – review and editing; Visualization; Methodology, **Stacie Loisate:** Formal analysis; Writing – original draft; **Amy Winter:** Resources; Writing – original draft **William J. Moss:** Writing – original draft; Resources, **John R Giles:** Resources; Investigation^,^ **C.J.E. Metcalf:** Writing – original draft; Methodology; Conceptualization, **Simon Mutembo:** Conceptualization; Resources, **Amy Wesolowski:** Writing – original draft; Writing – review and editing; Supervision; Funding acquisition; Formal analysis.

## Declaration of Competing Interest

None. This research did not receive any specific grant from funding agencies in the public, commercial, or not-for-profit sectors.
