# Supplementary material for: Impact of disruptions to routine vaccination programs, quantifying burden of measles, and mapping targeted supplementary immunization activities
Source: Epidemics. 2022 Dec;41:100647. doi: 10.1016/j.epidem.2022.100647 (PMC9742850; doi:10.1016/j.epidem.2022.100647)
Supplement: Supplementary file 1 — Supplementary material [file mmc1.pdf]

Supplementary Information

Table S1. Model initialization values and key parameters

Parameter	Value	Comments
Gravity model		
Θ (mean, sd)	0.000428 (4.485e-5)	Proportionality constant
ω_1 (mean, sd)	0.610 (5.099e-2)	Weight modifying contribution of population at origin [28]
ω_2 (mean, sd)	0.754 (5.736e-2)	Weight modifying contribution of population at destination [28]
γ (mean, sd)	0.519 (3.208e-4)	Weight modifying contribution of distance [28\
TSIR model		
β_1	0.3	Variation of amplitude [30]
β_0	15	Transmission coefficient [29]
α	0.975	Mixing parameter [31]
Vaccine efficacy	93%	[33]
2016 SIA coverage	95%	[32]
2020 SIA coverage	75%	[38]
Travel scaling factor	0.3	Proportion by which trips are scaled by to capture children's travel

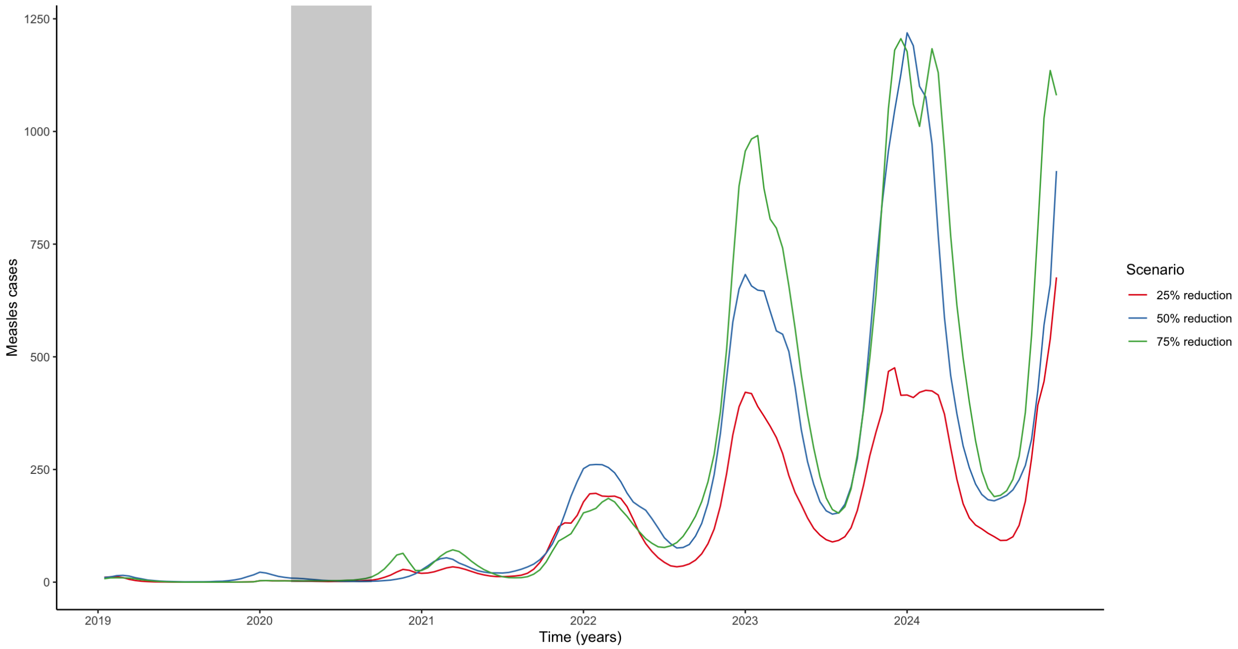

Figure S1. Time series of measles burden post-disruptions lasting six months with varying magnitudes. Disruptions of 25%, 50%, or 75% all produced increased measles burden nationally.

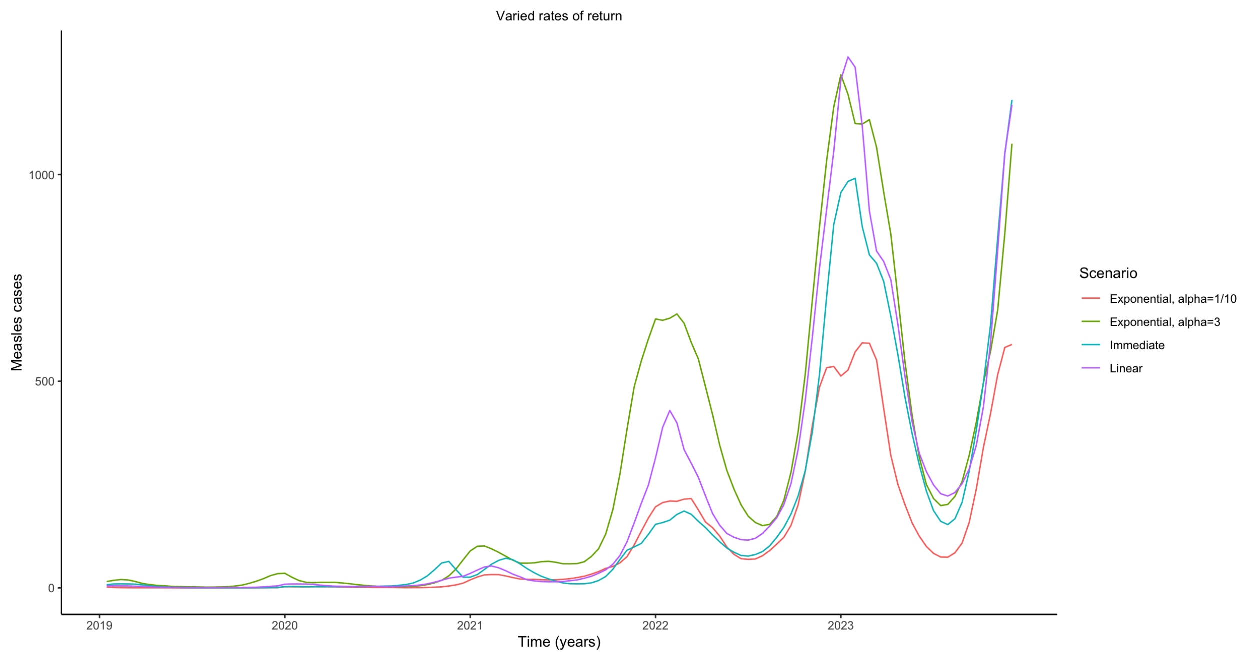

Figure S2. National measles burden considering varied rates of return to routine MCV1 coverage post disruptions lasting six months. Faster rates of return to routine (immediate and exponential with alpha = 1/10) results in fewer overall measles cases. Slower rates of return (linear and exponential with alpha = 3) have greater burden of measles. It is imperative to focus efforts on getting routine MCV1 coverage back to pre-disruptions rates.

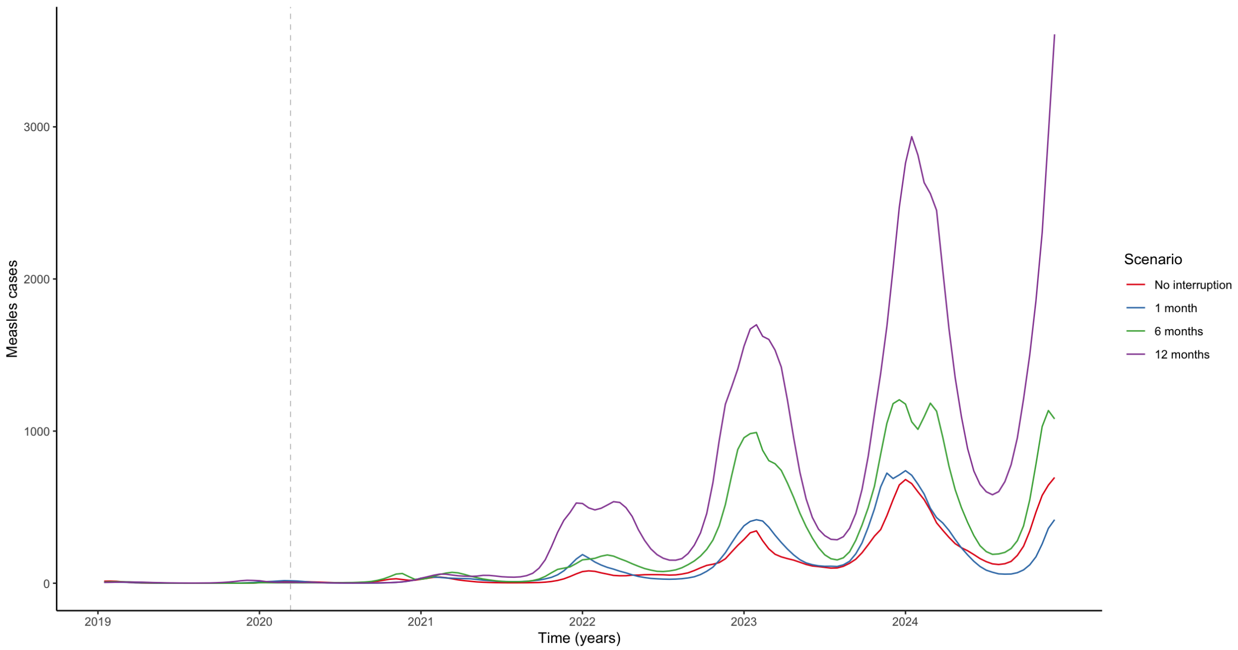

Figure S3. Time series of measles burden post-disruptions lasting one to six months. Disruptions start in March 2020 (gray dashed line) and last variable lengths of time. Increased periods of disruptions lead to increased measles burden nationally following disruptions.

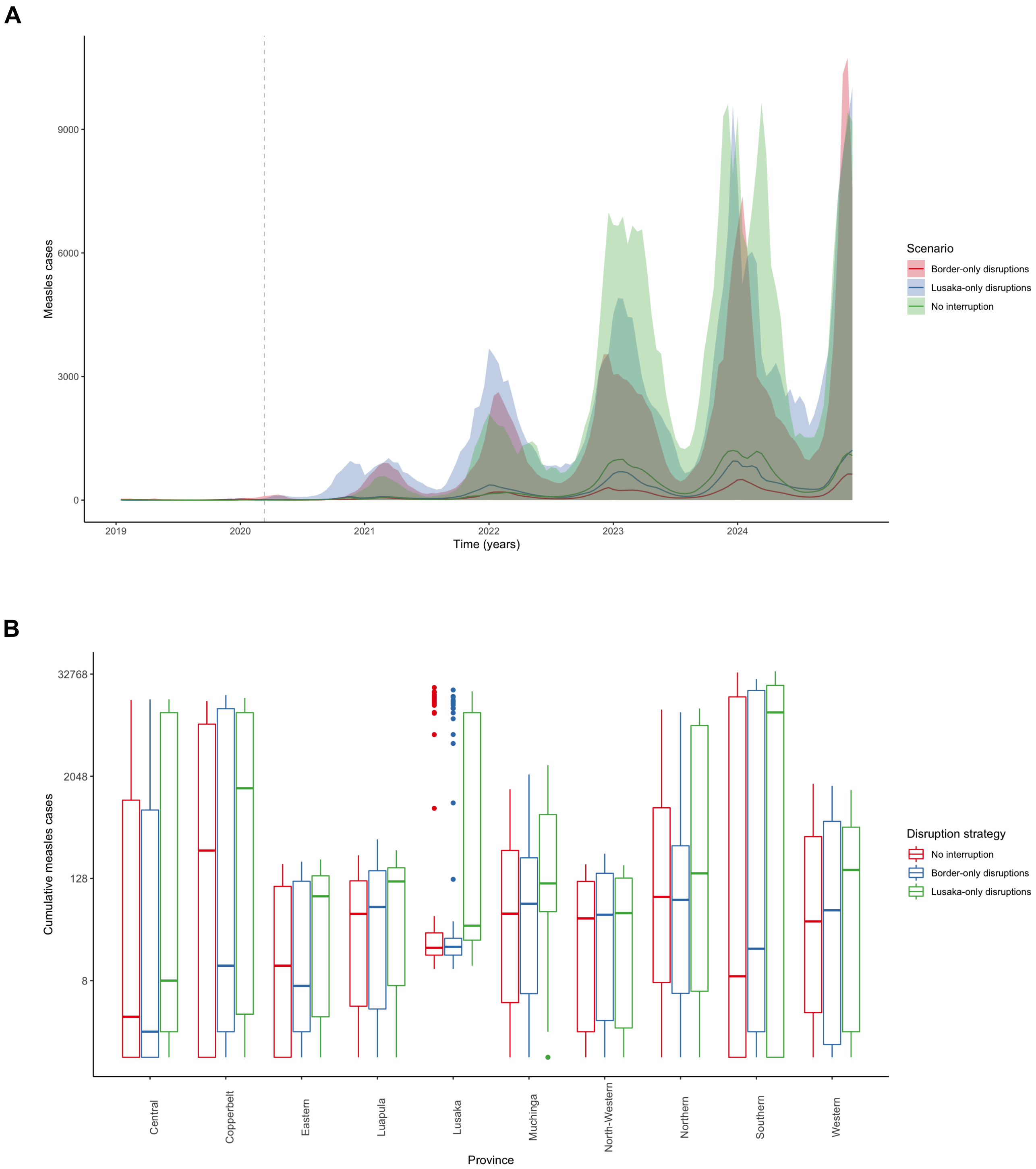

Figure S4. (a) Time series of measles cases under different disruption strategies. Border districts: Nakonde (Muchinga Province), Ndola and Chilabombwe (Copperbelt Province), and Chirundu (Lusaka Province). (b) Cumulative measles cases from January 2020 to December 2022 under different disruption strategies. Both scenarios (border and Lusaka-only) were not followed by an SIA.

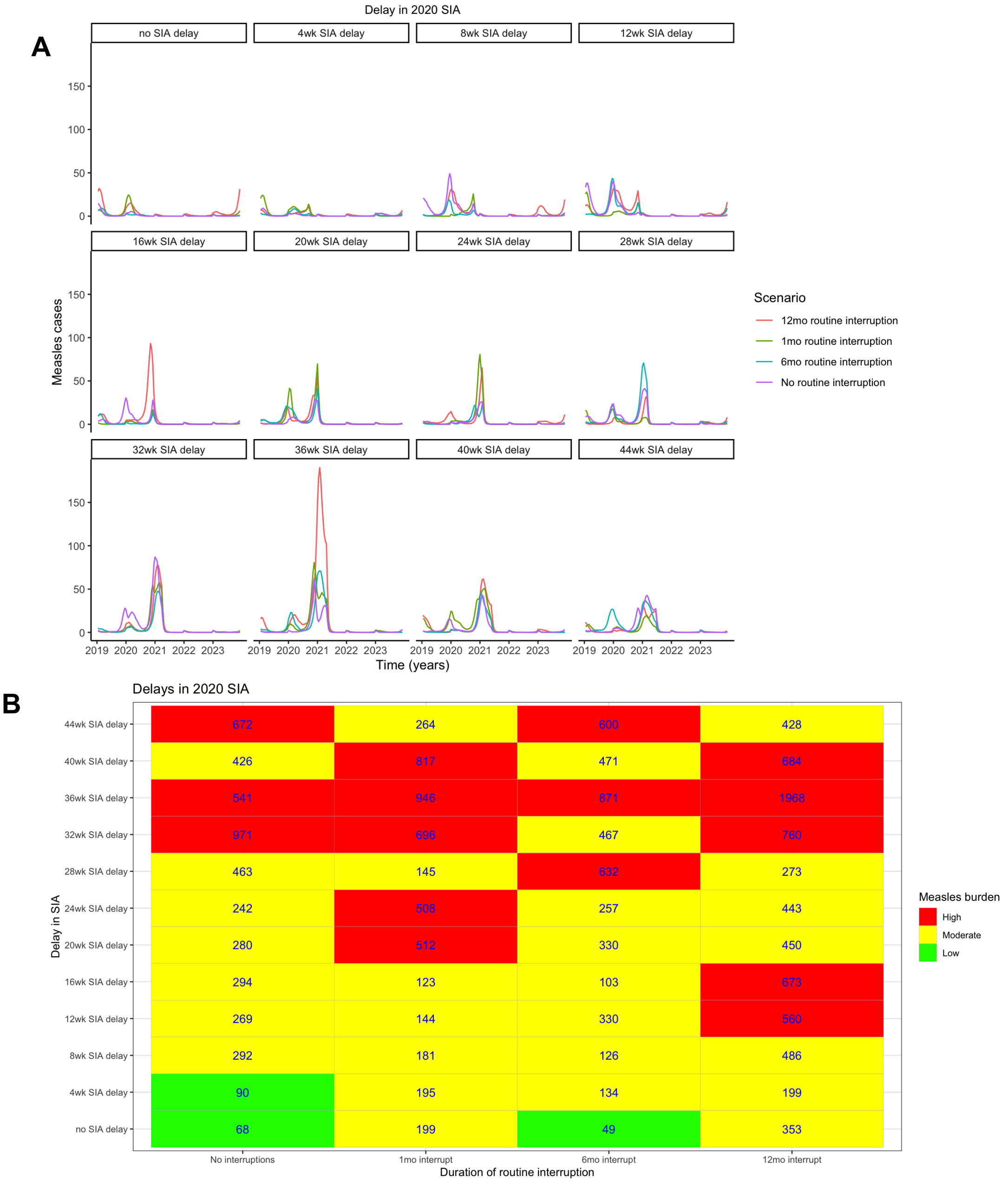

Figure S5. Delays in 2020 SIA implementation (a) Estimated measles burden. 2020 SIA is implemented with various delays in implementation and 75% coverage. (b) Cumulative estimated measles burden from January 2020 to December 2022, nationwide, under different delays in SIA (y-axis) and duration of disruptions in routine MCV1 (x-axis). Blue numbers represent average number of measles cases expected during this period.

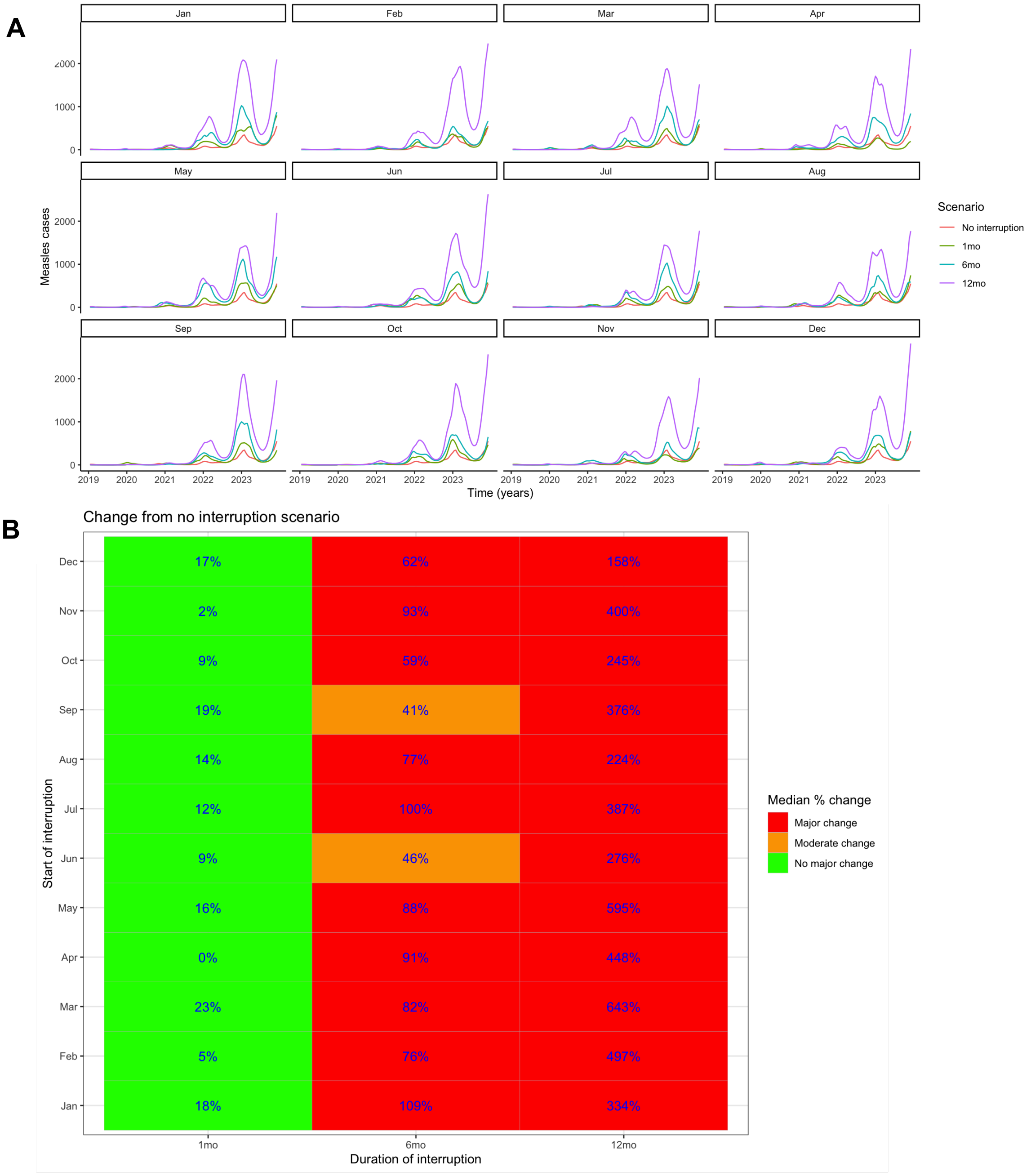

Figure S6. Beginning of disruption period (a) Estimated measles burden. Interruption scenarios (no interruption, 1 month disruption in routine MCV1, 6 months disruption in routine MCV1, and 12 months disruption in routine MCV1), starting at different months of the year. (b) Median percent change in cumulative number of cases of measles from January 2020 to December 2022 for disruption scenarios starting each calendar month of the year, compared to no interruption scenario. Values are colored based on the amount of change (no major change - <25% change; moderate change – 25-<50% change; major change – 50%+ change).

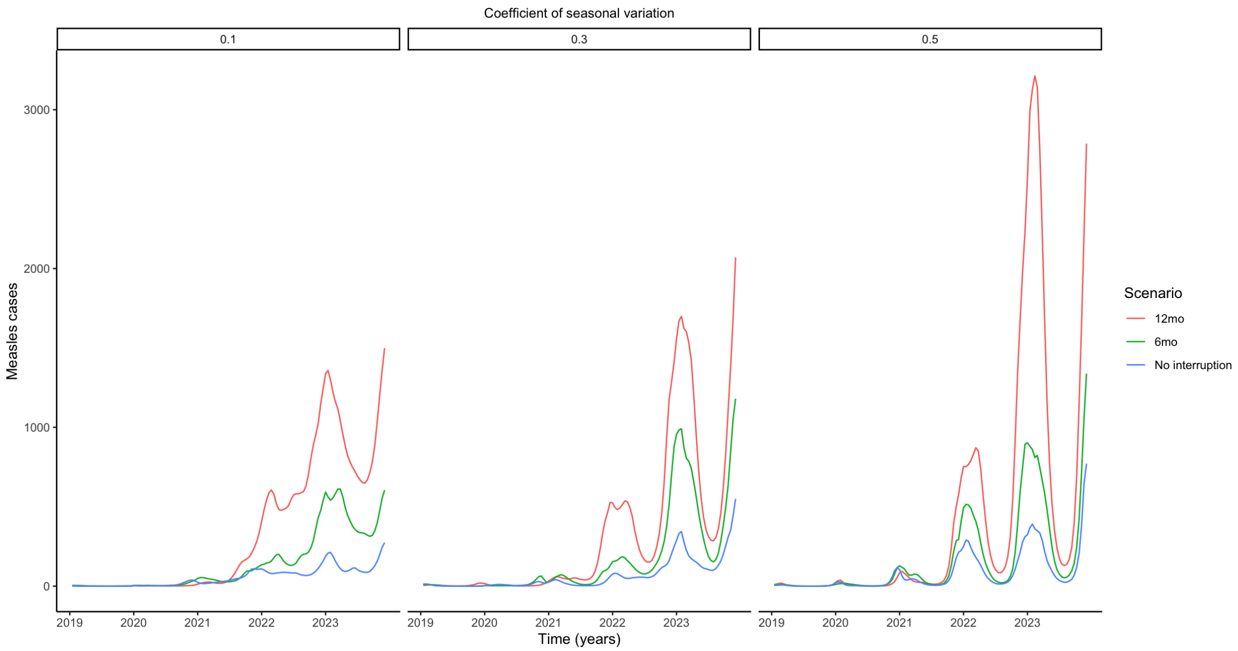

Figure S7. Variation in seasonal forcing coefficient. Estimated average number of measles cases for scenarios of different duration of interruption of routine services (no interruption, 6 months, and 12 months) for different values of coefficient of seasonal variation.

Table S2a. Classification of districts by level of expected measles burden following implementation of different SIA Strategies and under differing lengths of delay in SIA. Routine services are interrupted for 6 months, starting March 2020. Districts are classified as "Low" burden if they have <10 cases of measles from January 2020 to December 2022; "Moderate" if they have 10 – 49 cases of measles during this period, and "High" if they have 50 or more cases of measles.

Delay in SIA	SIA strategy	Expected measles burden	Districts
6 months	High Risk Provinces only	High	8
		Low	102
		Moderate	5
	National	High	5
		Low	107
		Moderate	3
No delay	High Risk Provinces only	High	4
		Low	106
		Moderate	5
	National	High	1
		Low	110
		Moderate	4

Table S2b. Distribution of district-level expected measles burden following disruptions of varying lengths of one month, six months, or one year. National 2020 SIA is implemented.

Length of disruption	Expected measles burden	Districts
1mo	High	12
	Low	98
	Moderate	5
1yr	High	22
	Low	22
	Moderate	71
6mo	High	14
	Low	79
	Moderate	22
